# Alcohol Consumption and Digestive Cancer Mortality in Koreans: The Kangwha Cohort Study

**DOI:** 10.2188/jea.JE20090077

**Published:** 2010-05-05

**Authors:** Sang-Wook Yi, Jae Woong Sull, John Alderman Linton, Chung Mo Nam, Heechoul Ohrr

**Affiliations:** 1 Department of Preventive Medicine and Public Health, Kwandong University College of Medicine, Gangneung, Korea; 2 Institute for Health Promotion, Graduate School of Public Health, Yonsei University, Seoul, Korea; 3 Department of Family Medicine, Yonsei University College of Medicine, Seoul, Korea; 4 Department of Preventive Medicine and Public Health, Yonsei University College of Medicine, Seoul, Korea

**Keywords:** alcohol consumption, digestive cancer, cohort study, mortality

## Abstract

**Background:**

Alcohol consumption is a known risk factor for cancers of the mouth, esophagus, liver, colon, and breast. In this study, we examined the association between alcohol consumption and digestive cancer mortality in Korean men and women.

**Methods:**

A cohort of 6291 residents of Kangwha County who were aged 55 years or older in March 1985 were followed to 31 December 2005—a period of 20.8 years. We calculated the relative risks of cancer mortality with respect to the amount of alcohol consumed. Cox proportional hazard model was used to adjust for age at entry, smoking, ginseng intake, education status, and pesticide use.

**Results:**

In men, the risks of mortality from esophageal cancer (relative risk [RR], 5.62; 95% confidence interval [CI], 1.45–21.77) and colon cancer (RR, 4.59; 95% CI, 1.10–19.2) were higher among heavy drinkers, as compared with abstainers. The risks of mortality from colon cancer and bile duct cancer rose with increasing alcohol consumption; these trends were positive and statistically significant (*P* = 0.04 and *P* = 0.02, respectively). When participants were stratified by type of alcoholic beverage, *soju* drinkers had higher risks of mortality from esophageal cancer and colon cancer than *makkoli* drinkers. In women, the risk of digestive cancer mortality was higher among alcohol drinkers than abstainers, but this difference was not statistically significant.

**Conclusions:**

Alcohol consumption increases mortality from esophageal cancer and colon cancer in men.

## INTRODUCTION

The proportion of Korean adults who drink alcohol is among the world’s highest^1^ and is increasing—from 45.8% in 1989 to 59.2% in 2005.^2^ According to The Third Korea National Health and Nutrition Examination Survey (NHANES) in 2005, in which a heavy drinker was defined as a person who drinks more than 6 glasses or 60 grams of *soju* “for men” or more than 4 glasses or 40 grams of *soju* “for women” at least once a week, 46.3% of men and 9.2% of women were heavy drinkers in Korea.^2^ *Soju* is a distilled alcoholic beverage native to Korea, and is similar to liquor or Japanese *shochu*; *makkoli* is an unfiltered alcoholic beverage, also native to Korea. In 1985, at the time the Kangwha Cohort survey began, the pure alcohol content was 25% for *soju* and 6% for *makkoli*.^3^^,^^4^

Numerous studies have shown that alcohol consumption is associated with risks of oral cancer, esophageal cancer, liver cancer, colon cancer, and breast cancer.^5^^–^^8^ However, a few studies reported no relation between alcohol consumption and digestive cancer.^9^ Other studies have shown that the relation between alcohol consumption and cancer differs with the type of alcohol consumed.^10^^–^^12^

Although a very large number of studies on the relation between alcohol consumption and the risk of cancer have been conducted in various countries, there have been few such studies of Korean populations, among whom the prevalence of drinking is quite high. We therefore examined the association between alcohol consumption and mortality risk from digestive cancer over a 20-year follow-up period in a Korean population, the Kangwha cohort. We also explored whether the association differed according to the type of alcoholic beverage consumed, as was the case in previous studies.

## METHODS

### Study population

This study used data from the Kangwha Cohort, which was formed in March 1985.^13^ Kangwha County consists of several islands approximately 50 km west from Seoul. The population of the county was 71 116 in 1993.^14^ The number of Kangwha County residents aged 55 years or older in February 1985 was 9378. Among them, 67.9%, or 6372 residents, participated in the interview and measurements of blood pressure and body mass. All those with missing information on alcohol intake at entry (*n* = 51) or who were not followed up after the initial survey (*n* = 30) were excluded. Thus, the final study population recruited was 6291 (2696 men, 3595 women). The Institutional Review Board of Human Research of Yonsei University approved the study (Approval No. 4-2007-0182).

### Baseline data collection and follow-up

The primary survey for the Kangwha Cohort was conducted over 1 month in March 1985. Each subject was interviewed using a structured questionnaire requesting information on demographic characteristics, including education, occupation, health condition at baseline, health behaviors, diet, and other factors. Blood pressure, height, and weight were measured by trained investigators. With regard to chronic disease, study subjects were asked to answer yes or no to the question, “Do you have any chronic disease, past accident, or injury that makes you uncomfortable in your daily life, including work?” If the participant answered yes, trained staff asked the subject about the type of disease or injury and recorded the relevant data. A total of 801 subjects reported neuralgia, 304 dyspepsia, 655 hypertension, 134 stroke, 104 arthritis, 104 diabetes mellitus, 287 dyspnea, 75 traffic accident, 94 lumbago, and 335 reported other conditions. In the present analysis, however, only information on the presence of chronic disease (ie, yes or no) was used as a covariate for analysis. The study subjects were followed up until 31 December 2005; thus, the maximum period of follow-up was 20.8 years.

### Outcome assessment

Deaths among subjects from 1 January 1992 through 31 December 2005 were confirmed by matching information with death records from the National Statistical Office. Follow-up was performed through record linkage at the national level and was complete, except for the case of emigrants. Data for those who died from 15 March 1985 through 31 December 1991 were collected either through calls or visits of trained surveyors twice a year or from records of burial and death certificates at *eup* and *myeon* offices, which are administrative branch offices of local governments in Korea.

The main outcome variables for this study were cause-specific cancer deaths as defined by The International Classification of Disease, 10th edition (C00–C99).

### Estimation of alcohol consumption

Study participants were asked to answer the question, “Do you drink alcohol” (yes or no). The frequency of drinking was recorded as daily, almost daily, 2 to 3 times a week, 1 to 4 times a month, or 4 to 12 times a year. The question regarding the type of alcoholic beverage and the amount of alcohol consumed was, “How much (in bottles or glasses) do you drink of a type of alcoholic beverage?” Participants were also asked to provide a numerical answer to the question, “When did you start drinking? Answer: From __ years ago (from __ years of age).”

Pure alcohol content and the bottle volume of each type of alcoholic beverage were determined based on data from the year 1985.^3^ Weekly alcohol consumption (grams) was estimated by multiplying the amount of alcohol consumed on 1 occasion (mL) by the pure alcohol content in the particular type of alcoholic beverage, the frequency of drinking per week, and the specific gravity of alcohol. A different weight was given to each frequency of drinking (daily: 7.0; almost daily: 5.5; 2–3 times a week: 2.5; 1–4 times a month: 0.625; 4–12 times a year: 0.163; nondrinking: 0).^15^ Alcohol was assumed to have a specific gravity of 0.785. When a participant reported consuming 2 types of alcoholic beverage, and both types of alcohol were consumed on 1 occasion, the weekly alcohol consumption for each type was estimated and the larger alcohol consumption value was used in the analysis.

### Statistical analysis

Study participants were divided into drinkers and nondrinkers and analyzed for the association between alcohol consumption and the risk of cancer mortality. They were further divided by the amount of alcohol consumed, in order to examine the dose-response relationship among subgroups. The male drinking group was subdivided into tertiles: low alcohol consumption (<138 g/week), moderate alcohol consumption (<540 g/week), and high alcohol consumption (≥540 g/week), whereas the female drinking group, which was small, was subdivided by the median into low alcohol consumption (<12 g/week) and high alcohol consumption (≥12 g/week) subgroups. Because there might be a difference in the risk of mortality between men and women, all analyses were stratified by sex. We used Cox proportional hazard model to test whether mortality risk—after adjustment for age (continuous), history of disease (ever, never), smoking habit (never, past, current smoker), ginseng intake (none, rarely, often, very often), pesticide use (user, nonuser), body mass index, and education status (none, elementary school, and high school)—differed according to the amount of alcohol consumed. All analyses were performed with SAS version 9.1 for Microsoft Windows.

## RESULTS

The sociodemographic characteristics of alcohol drinkers and nondrinkers are shown in Table 1. The majority of participants were older than 65 years; the mean (standard deviation) age of men and women was 66.3 (7.2) and 66.9 (7.1), respectively. On average, alcohol drinkers were younger than nondrinkers. Smoking was associated with alcohol drinking, ie, alcohol drinkers, both male and female, were more likely to be current smokers. Pesticide use was also associated with alcohol drinking.

**Table 1.** Baseline characteristics of alcohol drinkers and nondrinkers in the Kangwha cohort (1985–2005)

Characteristics	Men			Women		
	
	Drinkers (*n* = 1757)	Nondrinkers (*n* = 939)	*t* or χ^2^ value	Drinkers (*n* = 364)	Nondrinkers (*n* = 3231)	*t* or χ^2^ value
	Mean ± SD	Mean ± SD		Mean ± SD	Mean ± SD	
		
Age, years	65.7 ± 6.9	67.4 ± 7.8	5.44^a^	66.6 ± 7.9	67.1 ± 8.6	1.09
	
Body mass index, kg/m^2^	22.6 ± 20.3	22.8 ± 23.3	0.28	23.6 ± 24.6	24.7 ± 31.4	0.77

	*n* (%)	*n* (%)		*n* (%)	*n* (%)	
		
Chronic disease			0.34			3.76
Ever	790 (45.0)	434 (46.2)		200 (55.0)	1602 (49.6)	
Never	967 (55.0)	505 (53.8)		164 (45.0)	1629 (50.4)	
Education			2.693			0.43
None	721 (41.0)	372 (39.7)		296 (81.3)	2609 (80.8)	
Elementary	906 (51.6)	480 (51.2)		65 (17.9)	582 (18.0)	
High	130 (7.4)	86 (9.1)		3 (0.8)	39 (1.2)	
Smoking			105.7^a^			222 ^a^
Current	1409 (80.2)	593 (63.2)		194 (53.3)	613 (19.0)	
Never	228 (13.0)	267 (28.4)		162 (44.5)	2546 (78.8)	
Former	120 (6.8)	79 (8.4)		8 (2.2)	72 (2.2)	
Ginseng intake			6.8			9.04^b^
None	520 (29.7)	320 (34.4)		193 (54.2)	1552 (48.4)	
Rarely	875 (49.9)	435 (46.7)		128 (36.0)	1389 (43.3)	
Often	284 (16.2)	135 (14.5)		24 (6.7)	207 (6.5)	
Very often	73 (4.2)	41 (4.4)		11 (3.1)	58 (1.8)	
Pesticide use			11.5^a^			4.4^b^
User	1209 (68.8)	584 (62.3)		83 (22.8)	590 (18.3)	
Nonuser	548 (31.2)	354 (37.7)		281 (77.2)	2641 (81.7)	

^a^*P* < 0.01.

^b^*P* < 0.05.

During the 20.8 years of follow-up, 227 men and 128 women died from digestive cancer. Table 2 shows the relative risks of mortality from site-specific cancers in men and women. As compared with male nondrinkers, male alcohol drinkers had elevated risks of mortality from cancers of the esophagus, colon, bile duct, and pancreas but, with the exception of pancreatic cancer, these risks were not statistically significant. In women, alcohol drinkers had a higher risk of mortality from bile duct cancer than did nondrinkers (RR, 7.39; 95% CI, 1.71–31.9).

**Table 2.** Number of deaths and adjusted^a^ relative risks of death from all digestive cancers and site-specific cancers among alcohol drinkers in the Kangwha cohort (1985–2005)

Type of cancer	ICD 10	Men		Women	
	
		No. of Deaths	RR (95% CI)	No. of Deaths	RR (95% CI)
All digestive cancers	C15–C26	230	1.23 (0.92–1.64)	130	1.40 (0.83–2.36)
Esophageal cancer	C15	19	2.86 (0.82–10.1)	3	—
Stomach cancer	C16	99	1.11 (0.72–1.71)	52	1.64 (0.75–3.62)
Liver cancer	C22	36	0.83 (0.41–1.65)	19	2.13 (0.65–6.98)
Colon cancer	C18	17	2.85 (0.80–10.2)	12	—
Rectal cancer	C19–20	9	0.94 (0.23–3.87)	4	2.35 (0.19–28.6)
Colorectal cancer	C18–20	26	1.87 (0.74–4.76)	16	0.65 (0.08–5.40)
Bile duct cancer	C24	9	2.11 (0.43–10.4)	8	7.39 (1.71–31.9)
Pancreatic cancer	C25	15	7.90 (1.03–60.6)	14	—

^a^Adjusted for age (year of recruitment), history of chronic disease, smoking habit, ginseng intake, pesticide use, body mass index, and education status, using the Cox proportional hazard model.

Abbreviations: RR, relative risk; CI, confidence interval.

Table 3 shows the male participants’ relative risks of mortality from all digestive cancers and site-specific cancers, according to the amount of alcohol consumed (none, low, moderate, high). As compared with no alcohol consumption, high alcohol consumption was associated with a significantly higher risk of mortality from esophageal cancer and colon cancer: the relative risk (95% CI) of mortality was 5.62 (1.45–21.77) for esophageal cancer and 4.59 (1.10–19.20) for colon cancer. In cases of colon cancer and bile duct cancer, the risk of mortality significantly increased with rising frequency of drinking (*P* for trend = 0.04 and 0.02, respectively).

**Table 3.** Number of deaths and adjusted^a^ relative risks of death from all digestive cancers and site-specific cancers among men, by amount of alcohol consumed weekly

Type of cancer	Alcohol consumption				

	None (*n* = 947)	Low (<138 g/week) (*n* = 650)	Moderate (<540 g/week) (*n* = 538)	High (≥540 g/week) (*n* = 561)	*P* for trend
All digestive cancers					
No. of cases	71	60	44	55	
RR (95% CI)	1.00	1.18 (0.83–1.69)	1.06 (0.73–1.56)	1.26 (0.88–1.82)	0.26
Esophageal cancer					
No. of cases	3	3	4	9	
RR (95% CI)	1.00	1.04 (0.17–6.32)	2.45 (0.53–11.29)	5.62 (1.45–21.77)	0.09
Stomach cancer					
No. of cases	35	29	16	20	
RR (95% CI)	1.00	1.19 (0.71–1.99)	0.82 (0.45–1.50)	1.01 (0.57–1.77)	0.71
Liver cancer					
No. of cases	13	8	8	8	
RR (95% CI)	1.00	0.91 (0.38–2.22)	0.94 (0.38–2.28)	0.79 (0.31–2.01)	0.11
Colon cancer					
No. of cases	3	4	4	6	
RR (95% CI)	1.00	1.13 (0.19–6.83)	2.98 (0.65–13.7)	4.59 (1.10–19.2)	0.04
Rectal cancer					
No. of cases	3	4	0	2	
RR (95% CI)	1.00	1.86 (0.41–8.45)	—	1.01 (0.16–6.25)	0.75
Colorectal cancer					
No. of cases	6	8	4	8	
RR (95% CI)	1.00	1.57 (0.50–4.91)	1.33 (0.37–4.82)	2.61 (0.88–7.78)	0.14
Bile duct cancer					
No. of cases	3	2	2	3	
RR (95% CI)	1.00	1.67 (0.23–12.0)	2.01 (0.28–14.6)	3.06 (0.49–19.1)	0.02
Pancreatic cancer					
No. of cases	2	6	3	5	
RR (95% CI)	1.00	4.68 (0.94–23.4)	2.77 (0.46–16.9)	3.77 (0.68–21.0)	0.48

^a^Adjusted for age (year of recruitment), history of chronic disease, smoking habit, ginseng intake, pesticide use, body mass index, and education status, using the Cox proportional hazard model.

Abbreviations: RR, relative risk; CI, confidence interval.

Table 4 shows the results of the same analysis for women. An increased risk for all digestive cancers was observed in the high alcohol consumption subgroup, but the association was not statistically significant. However, there was a significantly elevated risk for stomach cancer in the high alcohol consumption subgroup.

**Table 4.** Number of deaths and adjusted^a^ relative risks of death from all digestive cancers and site-specific cancers among women, by amount of alcohol consumed weekly

Type of cancer	Alcohol consumption			

	None (*n* = 3234)	Low (<12 g/week) (*n* = 179)	High (≥12 g/week) (*n* = 182)	*P* for trend
All digestive cancers				
No. of cases	112	8	10	
RR (95% CI)	1.00	1.15 (0.53–2.51)	1.63 (0.83–3.19)	0.20
Stomach cancer				
No. of cases	45	2	6	
RR (95% CI)	1.00	0.86 (0.20–3.60)	2.59 (1.06–6.33)	0.38
Bile duct cancer				
No. of cases	15	3	1	
RR (95% CI)	1.00	8.21 (1.53–43.9)	7.01 (0.77–63.6)	0.43
Liver cancer				
No. of cases	5	2	1	
RR (95% CI)	1.00	3.49 (0.94–13.0)	1.06 (0.13–8.47)	0.99

^a^Adjusted for age “year of recruitment” the history of chronic disease, smoking habits, ginseng intake, pesticide use, body mass index, and education status, using the Cox proportional hazard model.

Abbreviations: RR, relative risk; CI, confidence interval.

We conducted a stratified analysis by type of alcoholic beverage consumed by men. Table 5 shows the relative risks of mortality from all digestive cancers and site-specific cancers by amount of alcohol consumed among male *soju* drinkers. In the high alcohol consumption subgroup, mortality risks for esophageal cancer and colon cancer were greater than those for other digestive cancers: the RR (95% CI) was 6.98 (1.62–30.0) for esophageal cancer and 5.26 (1.10–25.2) for colon cancer. Among male *makkoli* drinkers, the associations were similar, but weaker (data not shown).

**Table 5.** Number of deaths and adjusted^a^ relative risks of death from all digestive cancers and site-specific cancers among male *soju*^b^ drinkers, by amount of alcohol consumed weekly

Type of cancer	Amount of alcohol consumed in the form of *soju*				

	None (*n* = 947)	Low (<138 g/week) (*n* = 315)	Moderate (<540 g/week) (*n* = 340)	High (≥540 g/week) (*n* = 341)	*P* for trend
All digestive cancers					
No. of cases	71	32	29	36	
RR (95% CI)	1.00	1.30 (0.85–1.99)	1.06 (0.68–1.65)	1.35 (0.89–2.05)	0.40
Esophageal cancer					
No. of cases	3	2	2	6	
RR (95% CI)	1.00	2.14 (0.35–13.0)	2.07 (0.33–12.9)	6.98 (1.62–30.0)	0.14
Stomach cancer					
No. of cases	33	15	9	14	
RR (95% CI)	1.00	1.28 (0.68–2.38)	0.69 (0.32–1.45)	1.15 (0.60–2.19)	0.93
Liver cancer					
No. of cases	13	6	7	6	
RR (95% CI)	1.00	1.28 (0.48–3.42)	1.23 (0.48–3.13)	0.91 (0.32–2.61)	0.77
Colon cancer					
No. of cases	3	2	2	4	
RR (95% CI)	1.00	2.14 (0.35–13.2)	2.27 (0.36–14.2)	5.26 (1.10–25.2)	0.08
Rectal cancer					
No. of cases	3	1	0	1	
RR (95% CI)	1.00	0.90 (0.09–8.94)	—	0.81 (0.08–8.28)	0.33
Colorectal cancer					
No. of cases	6	3	2	5	
RR (95% CI)	1.00	1.49 (0.37–6.11)	1.01 (0.20–5.15)	2.77 (0.80–9.54)	0.25
Bile duct cancer					
No. of cases	3	0	1	2	
RR (95% CI)	1.00	—	1.27 (0.11–14.9)	2.21 (0.28–17.5)	0.12
Pancreatic cancer					
No. of cases	2	3	3	3	
RR (95% CI)	1.00	4.53 (0.74–27.6)	4.20 (0.68–26.0)	4.26 (0.68–26.6)	0.27

^a^Adjusted for age (year of recruitment), history of chronic disease, smoking habit, ginseng intake, pesticide use, body mass index, and education status, using the Cox proportional hazard model.

^b^*Soju* is a distilled alcoholic beverage native to Korea, and is similar to liquor or Japanese *shochu*.

Abbreviations: RR, relative risk; CI, confidence interval.

## DISCUSSION

In an analysis of data obtained from the Kangwha Cohort, comprising people aged 55 years or older in 1985, there was no significant association between alcohol consumption and the risk of mortality from all digestive cancers. However, there were associations between alcohol consumption and site-specific cancer mortality from esophageal, colon, and pancreatic cancers.

The risk of mortality from esophageal cancer markedly increased among men as alcohol consumption rose; the risk was significantly elevated in the high alcohol consumption subgroup. These results confirm those of recent studies reporting that alcohol drinkers had a higher risk of mortality from esophageal cancer than nondrinkers.^7^^,^^16^ It has been reported that acetaldehyde, a highly reactive and toxic alcohol metabolite, is the carcinogen in alcohol that is responsible for esophageal cancer.^17^ Acetaldehyde interferes with DNA repair machinery and directly inhibits O6 methyl-guanyltransferase, an enzyme important for DNA repair. Inhalation of acetaldehyde can cause bronchial cancer, as well as esophageal cancer. In addition, 2 recent Japanese studies reported that alcohol consumption and smoking have a synergistic effect on esophageal cancer.^16^^,^^18^

There is controversy regarding the effect of alcohol on colon cancer risk. A recent cohort study of 14 304 Koreans aged 65 or older reported no association between alcohol drinking and colon cancer.^19^ However, in a meta-analysis of 16 cohort studies, high alcohol intake was significantly associated with an increased risk of colon cancer.^8^ The risk of colon cancer rose 15% for every 100-gram increase in weekly alcohol intake. In the present study, the risk of mortality from colon cancer rose with increasing alcohol consumption among men; in the high alcohol consumption subgroup, the risk was significantly elevated.

The risk of pancreatic cancer mortality is also reportedly higher among men. In a cohort study of 17 633 US white men largely of Scandinavian and German descent who had responded to a mailed questionnaire in 1966 and were followed-up through 1986 for mortality, the relative risk of mortality from pancreatic cancer was 3.1 (95% CI, 1.2–8.0) in those who drank 10 or more drinks per month, although the dose-response trends among drinkers were not linear.^20^ In a case-control study of 200 patients with pancreatic cancer, however, the risk of pancreatic cancer was lower in current drinkers.^21^ In the present study, a dose-response trend for the risk of mortality from pancreatic cancer was not apparent among drinkers. Therefore, we are unable to draw any conclusions regarding the association between alcohol consumption and the risk of mortality from pancreatic cancer.

A large meta-analysis showed that heavy alcohol consumption increased the risk of liver cancer, although the association was not strong.^9^ A recent Japanese study reported that liver cancer risk was actually lower in a high alcohol consumption subgroup.^22^ In the present study, there was no association between alcohol consumption and the risk of mortality from liver cancer. One possible reason for these different results among studies is that hepatitis B infection was a confounding factor.^22^ In Korea, hepatitis B carriers accounted for 7% to 10% of the total population in 1980,^23^^,^^24^ and approximately 5% since the early 1990s.^25^ This is much higher than the 0.1% to 0.5% prevalence in the United States and Europe.^26^ Accordingly, the incidence of liver cancer caused by hepatitis B virus infection is also very high in Korea. A Korean study examined the etiologic factors of hepatocellular carcinoma in 1078 patients with hepatocellular carcinoma who visited the National Cancer Center from June 2001 to December 2003, and found that 800 (74.2%) had hepatitis B virus infection, 93 (8.6%) had hepatitis C virus infection, 74 (6.9%) had abused alcohol, and 111 (10.3%) had non-B, non-C, nonalcoholic cirrhosis.^27^

The lack of a lifetime drinking history in our study might be another explanation for our divergent findings. In a recent case-control study of a Canadian population, daily drinkers with 180 or more drink-years had a 7.9 times higher risk of liver cancer than never drinkers; however, the risk of liver cancer among daily drinkers was not significantly higher when drink-years were not included in the analysis.^28^

*Soju* drinkers had a higher risk of mortality from esophageal cancer and colon cancer than *makkoli* drinkers. In this study, *soju* drinkers accounted for 53.4% of male drinkers, *makkoli* drinkers for 44.2%, and beer and other alcohol drinkers for 2.4% (data not shown). Many studies have shown that the risk of cancer differs according to the type of alcoholic beverage. In a Danish study, wine drinking was not associated with upper digestive tract cancer, whereas drinking beer or spirits increased the risk.^10^ Another study suggested that alcohol concentration is a risk factor for oral cancer.^11^ In addition, the risk of colon cancer was reported to be slightly higher among beer drinkers than among wine drinkers.^12^^,^^29^

This study has several limitations. First, data on alcohol consumption were collected through a questionnaire distributed to the Kangwha Cohort of people aged 55 years or older. Thus, validity is a concern. However, when another Korean study used a questionnaire to collect data on alcohol consumption among elderly people in 1998, its reliability and validity were found to be high.^30^ In addition, a research team conducted a second interview/test with 3381 survivors in the Kangwha Cohort in 1994. The percentage agreement between drinking status data collected in 1985 and those collected in 1994 was 87% and Cohen’s kappa value was 0.697. Thus, there was substantial agreement between the 2 datasets. A second potential limitation is that alcohol consumption is likely to change over a follow-up period of 20 years. Indeed, there are many reasons to suppose that drinking patterns would change as a population ages. For example, those with comorbidities or illnesses may stop drinking. Because the present study relied on the baseline data only, caution is advised in interpreting the results. However, we do know that of 1034 drinkers in 1985, 694 (67.1%) still drank alcohol in 1994, and that out of 2147 nondrinkers in 1985, 205 (9.5%) were drinkers in 1994. The Spearman rank-correlation coefficient between the 1985 and 1994 datasets on drinking frequency was 0.41, indicating that drinking frequency had not greatly changed over 10 years. Third, follow-up of death records was different in the 1985–1991 and 1992–2005 periods. However, there were only 129 (35.7%) cancer deaths in 1985–1991, and even when the analysis was limited to data on cases from 1992 to 2005 only, the results were similar. Fourth, the validity of a cancer diagnosis on death certificates was not examined separately. Therefore, the validity of cancer diagnoses made in the late 1980s and early 1990s may be limited. However, any misclassification that occurred was most likely nondifferential with respect to alcohol consumption. Fifth, it is possible that alcohol consumption among patients with cancer or chronic diseases was underestimated, ie, that their alcohol consumption was greater before their illness. However, with regard to selection relative to health status, when subjects who died during the first 2 years of follow-up were excluded from the analysis, the results of the analysis were largely unaffected. Sixth, sample sizes were small, with a limited number of deaths in some analyses. Analyses of the association between *soju* drinking and cancer mortality may therefore have limited statistical power, due to the insufficient number of cases. Seventh, the drinking habits of women differed greatly from those of men.^31^ Because only 10.1% of women were drinkers and female heavy drinkers were scarce, we were unable to examine the relationship between alcohol consumption and mortality in women to the same extent that we had in men. Eighth, although alcohol is an established risk factor for liver cancer, no significant increase in the risk for liver cancer was observed in this study. As mentioned above, hepatitis B may be a confounding factor in this association. However, as information on hepatitis B infection was not collected in the primary survey, adjustment for this potential confounder was not possible. Ninth, a very rough classification of smoking (current, never, past smoker) was used in the present study. As the risk of cancer increases with the number of cigarettes smoked, there is the possibility of residual confounding.

In conclusion, as compared with a nondrinking group, the risk of mortality from esophageal cancer steadily increased in alcohol drinkers as alcohol consumption rose. The risk of mortality was significantly increased for colon cancer, and was higher for pancreatic cancer as well. However, because this study investigated people aged 55 years or older living in an agricultural community and only a small number of women were drinkers, further studies will need to be undertaken.

## References

[1] Koh KH Estimates of alcohol consumption based on pure alcohol content and their comparison with data of other countries . Health and Welfare Policy Forum. 2000;6:77–86 [in Korean]

[2] Ministry of Health and Welfare. The Third Korea National Health and Nutrition Examination Survey (KNHANES III), 2005 – Summary. 2005.

[3] Korea Alcohol and Liquor Industry Association. Journal of Korea Alcohol and Liquor Industry. Korea Alcohol and Liquor Industry Association. Seoul. 1986 [in Korean].

[4] Chung W Type of alcoholic beverage and high-risk drinking: how risky is beer drinking in Korea? Alcohol Alcohol. 2004;39:39–42 10.1093/alcalc/agh015 14691073

[5] Seitz HK , Pöschl G , Simanowski UA Alcohol and cancer . Recent Dev Alcohol. 1998;14:67–95 10.1007/0-306-47148-5_4 9751943

[6] Bongaerts BW , van den Brandt PA , Goldbohm RA , de Goeij AF , Weijenberg MP Alcohol consumption, type of alcoholic beverage and risk of colorectal cancer at specific subsites . Int J Cancer. 2008;123:2411–7 10.1002/ijc.23774 18752250

[7] Fan Y , Yuan JM , Wang R , Gao YT , Yu MC Alcohol, tobacco, and diet in relation to esophageal cancer: the Shanghai Cohort Study . Nutr Cancer. 2008;60:354–63 10.1080/01635580701883011 18444169 PMC2409004

[8] La Vecchia C Alcohol and liver cancer . Eur J Cancer Prev. 2007;16:495–7 10.1097/CEJ.0b013e3280145b5d 18090120

[9] Bagnardi V , Blangiardo M , La Vecchia C , Corrao G A meta-analysis of alcohol drinking and cancer risk . Br J Cancer. 2001;85:1700–5 10.1054/bjoc.2001.2140 11742491 PMC2363992

[10] Grønbaek M , Becker U , Johansen D , Tønnesen H , Jensen G , Sørensen TI Population based cohort study of the association between alcohol intake and cancer of the upper digestive tract . BMJ. 1998;317:844–7 9748175 10.1136/bmj.317.7162.844 PMC31093

[11] Huang WY , Winn DM , Brown LM , Gridley G , Bravo-Otero E , Diehl SR , Alcohol concentration and risk of oral cancer in Puerto Rico . Am J Epidemiol. 2003;157:881–7 10.1093/aje/kwg055 12746240

[12] Ferrari P , Jenab M , Norat T , Moskal A , Slimani N , Olsen A , Lifetime and baseline alcohol intake and risk of colon and rectal cancers in the European prospective investigation into cancer and nutrition (EPIC) . Int J Cancer. 2007;121:2065–72 10.1002/ijc.22966 17640039

[13] Hong JS , Yi SW , Kang HC , Ohrr H Body Mass Index and Mortality due to Cardiovascular Disease in South Korean Men: Kangwha Cohort Study . Ann Epidemiol. 2007;17:622–7 10.1016/j.annepidem.2007.03.004 17553697

[14] Kangwha County basic statistics of Incheon Metropolitan City. Incheon Metropolitan City. 2008 [in Korean].

[15] National Center or Health Statistics, Harlan WR, Hill AL, Schmouder RP, et al. Dietary intake and cardiovascular risk factors, Part I. Blood pressure correlates. Vital and Health Statistics. Series 11, No. 226. DHHS Pub. No. (PHS) 83-1676. Public Health Service. Washington. U.S. Government Printing Office, February 1983.

[16] Sakata K , Hoshiyama Y , Morioka S , Hashimoto T , Takeshita T , Tamakoshi A Smoking, alcohol drinking and esophageal cancer: findings from the JACC Study . J Epidemiol. 2005;15 Suppl 2:S212–9 10.2188/jea.15.S212 16127236 PMC8639047

[17] Homann N , Kärkkäinen P , Koivisto T , Nosova T , Jokelainen K , Salaspuro M Effects of acetaldehyde on cell regeneration and differentiation of the upper gastrointestinal tract mucosa . J Natl Cancer Inst. 1997;89:1692–7 10.1093/jnci/89.22.1692 9390538

[18] Ishikawa A , Kuriyama S , Tsubono Y , Fukao A , Takahashi H , Tachiya H , Smoking, alcohol drinking, green tea consumption and the risk of esophageal cancer in Japanese men . J Epidemiol. 2006;16:185–92 10.2188/jea.16.185 16951537 PMC7683705

[19] Lim HJ , Park BJ Cohort study on the association between alcohol consumption and the risk of colorectal cancer in the Korean elderly . J Prev Med Pub Health. 2008;41:23–9 [in Korean]. 10.3961/jpmph.2008.41.1.23 18250602

[20] Zheng W , McLaughlin JK , Gridley G , Bjelke E , Schuman LM , Silverman DT , A cohort study of smoking, alcohol consumption, and dietary factors for pancreatic cancer (United States) . Cancer Causes Control. 1993;4:477–82 10.1007/BF00050867 8218880

[21] Inoue M , Tajima K , Takezaki T , Hamajima N , Hirose K , Ito H , Epidemiology of pancreatic cancer in Japan: a nested case-control study from the Hospital-based Epidemiologic Research Program at Aichi Cancer Center (HERPACC) . Int J Epidemiol. 2003;32:257–62 10.1093/ije/dyg062 12714546

[22] Tanaka K , Tsuji I , Wakai K , Nagata C , Mizoue T , Inoue M , Research Group for the Development and Evaluation of Cancer Prevention Strategies in Japan. Alcohol drinking and liver cancer risk: an evaluation based on a systematic review of epidemiologic evidence among the Japanese population . Jpn J Clin Oncol. 2008;38:816–38 10.1093/jjco/hyn108 18945722

[23] Yoo KY , Park BJ , Ahn YO Seroepidemiology of Hepatitis B virus Infection in Healthy Korean Adults in Seoul . Korean J Prev Med. 1988;21:89–98 [in Korean]

[24] Chun BY , Kyeong LM , Kyeong RY The prevalence of hepatitis B surface antigen among Korean byliterature review . Korean J Epidemiol. 1992;14:70–8 [in Korean]

[25] Kim DS , Kim YS , Kim JY , Ahn YO A Study on the Seropositivity of HBsAg among Biennial Health Examinees: A Nation-wide Multicenter Survey . J Prev Med Pub Health. 2002;35:129–35 [in Korean]

[26] Harrison’s Principles of Internal Medicine. 15th ed. International ed. Vol. 2. Acute viral Hepatitis (Chapter 295). New York: McGraw-Hill Co.; 2001. p. 1729.

[27] Cheon JH , Park JW , Park KW , Kim YI , Kim SH , Lee WJ , The Clinical Report of 1,078 Cases of Hepatocellular Carcinomas: National Cancer Center Experience . Korean J Hepatol. 2004;10:288–97 15613804

[28] Benedetti A , Parent ME , Siemiatycki J Lifetime consumption of alcoholic beverages and risk of 13 types of cancer in men: results from a case-control study in Montreal . Cancer Detect Prev. 2009;32:352–62 10.1016/j.canep.2009.03.001 19588541

[29] Pedersen A , Johansen C , Grønbaek M Relations between amount and type of alcohol and colon and rectal cancer in a Danish population based cohort study . Gut. 2003;52:861–7 10.1136/gut.52.6.861 12740343 PMC1773681

[30] Park BJ , Kim DS , Koo HW , Bae JM Reliability and validity study of a life style questionnaire for elderly people . J Prev Med Pub Health. 1998;31:49–58 [in Korean]

[31] Plant ML. Women and alcohol: contemporary and historical perspectives. London: Free Association Books; 1997.

